# Controlled Seeding of β‑Amyloid Fibrillation Reveals Propagation of Structural Polymorphisms in Cellular Environments

**DOI:** 10.1021/acs.biochem.6c00013

**Published:** 2026-04-06

**Authors:** Tingyao Wang, Yan Sun, Yi Ran Lin, Lan Yao, Wei Qiang

**Affiliations:** † Department of Chemistry, 14787 Binghamton University, The State University of New York, Vestal, New York 13850, United States; ‡ Small Scale System Integration and Packaging (S3IP), Binghamton University, Vestal, New York 13850, United States

## Abstract

Molecular-level structural polymorphisms of β-amyloid (Aβ) aggregates in Alzheimer’s disease patients are pathologically important. However, tracking the propagation and modulation of Aβ structural polymorphisms via *ex vivo* approaches remains challenging. The successful application of cryogenic transmission electron microscopy (cryo-TEM) in this area relies on the availability of morphologically distinct micrographs, which then enable unambiguous three-dimensional structural reconstruction of individual fibrillar polymorphs to achieve optimal resolution. As a complementary approach, solid-state nuclear magnetic resonance (ssNMR) spectroscopy with guided isotope-labeling schemes can provide site-specific and quantitative information on the populations of individual polymorphs. Such ssNMR sample preparations require *ex vivo* seeding, in which key parameters—including seed concentration and seeding time—must be carefully controlled for individual Aβ-cell systems to avoid the introduction of self-nucleated fibrillar polymorphs. In this work, we show that the application of controlled *ex vivo* seeding combined with quantitative ssNMR spectroscopy reveals the propagation of molecular-level structural polymorphs, depending on the types of seeds and cells.

## Graphical abstract

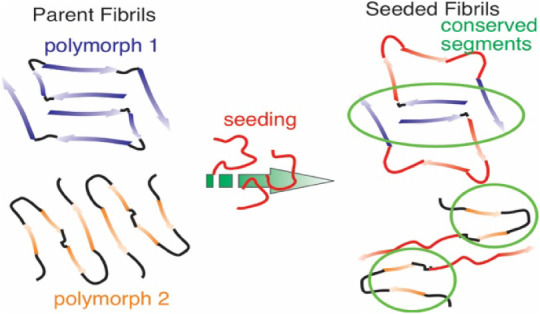

Deposition of extracellular β-amyloid (Aβ) plaques is a clinical hallmark of Alzheimer’s disease (AD).^1,2^ Molecular-level structural polymorphisms of fibrillar Aβ aggregates within these plaques are considered a pathologically relevant factor in AD.^3,4^ This hypothesis was supported by *ex vivo* structural characterization of Aβ fibrillar polymorphisms in AD brain-tissue-seeded fibrils using solid-state nuclear magnetic resonance (ssNMR) spectroscopy.^5,6^ These works demonstrated a positive correlation between Aβ_40_ (the 40-residue Aβ isoform) fibrillar structural polymorphism in *ex vivo*-seeded fibrils and AD progression and patients’ clinical histories.^5^ With the rapid development of single-particle cryogenic transmission electron microscopy (cryo-TEM), understanding of brain-extracted Aβ fibrillar polymorphisms has expanded significantly over the past decade. For instance, cryo-TEM characterization of brain-tissue-extracted Aβ_40_ and Aβ_42_ (the 42-residue Aβ isoform) have yielded atomic-level structural models at ∼2.5 Å resolution.^7–11^ These advances clearly demonstrated distinct Aβ fibrillar polymorphisms between brain-extracted and *in vitro* filaments, as well as among fibrils extracted from different AD clinical subtypes.

However, applications of cryo-TEM in the study of Aβ fibrillar polymorphisms and the propagation of molecular structures through seeded fibrillation remain challenging. Establishing high-resolution cryo-TEM-based structures for individual structural polymorphs relies on the availability of a large number of high-quality micrographs, which can be difficult to obtain for minor polymorphs. In addition, it is common to achieve lower-resolution structural reconstructions for fibrillar segments that exhibit greater structural disorder. Quantitative ssNMR spectroscopy provides a complementary approach to address these challenges. With guidance from cryo-TEM-based models of individual polymorphs (which may be of lower resolution), selective isotope labeling can be introduced at specific sites of interest. However, unlike cryo-TEM samples, which utilize fibrils extracted directly from brain tissue, isotope-labeled ssNMR samples at the milligram scale require *ex vivo* seeding.^6,12^ This may introduce biased structural polymorph populations that dampen the correlation between cryo-TEM and ssNMR-based characterizations. It is crucial to optimize seeding procedures to eliminate such biases.

We previously described a controlled *ex vivo* seeding protocol that enables the elimination of self-nucleation-driven fibrillation during seeding procedures.^13^ This protocol involves quantification of Aβ concentrations in extracted *ex vivo* seeds using SDS-PAGE, followed by optimization of seeding times using Thioflavin-T (ThT) kinetics assays. In the present work, we applied this approach to the *ex vivo* seeding of a pathologically relevant post-translationally modified (PTM) Aβ variant, namely the serine-8-phosphorylated Aβ_40_ (pS8-Aβ_40_). Seeding was performed in both mouse and human neuroblastoma cell lines (Neuro2a and SH-SY5Y respectively) to investigate the influence of cell type on Aβ fibrillar polymorphisms using quantitative ssNMR methods.

## Materials and Methods

### Peptide Synthesis and Purifications

All Aβ_40_ peptides, including wild-type Aβ_40_ and the pS8-Aβ_40_ variant with and without isotope labeling, were synthesized on a microwave-assisted automated peptide synthesizer (Biotage Initiator^+^ Alstra) using a standard Fmoc chemistry and previously described protocols.^14^ Isotope-labeled amino acids (Cambridge Isotope Inc.) were incorporated at specific sites during synthesis. Crude peptides were purified by high-performance liquid chromatography (HPLC, Agilent 1260) using a C18 reversed-phase column and a linear water–acetonitrile gradient. All peptides were confirmed to have >95% purity by LC–MS analysis (Shimadzu LCMS 2020).

### Cell Culturing and Amyloid Extraction

Culturing of Neuro2a (N2a, ATCC) and SH-SY5Y (ECACC) cells followed previously developed protocols.^13,15^ Briefly, N2a cells were cultured in a 1:1 *v/v* mixture of Dulbecco’s modified Eagle’s medium (DMEM, Sigma-Aldrich) and Minimum Essential Medium Eagle (MEM, Sigma-Aldrich), supplemented with 10% fetal bovine serum (FBS, Sigma-Aldrich) and 2% penicillin-streptomycin (Pen-Strep, 10,000 units/mL penicillin and 10,000 μg/mL streptomycin, Gibco). SH-SY5Y cells were cultured in a 1:1 DMEM and Ham’s F12 medium (Sigma-Aldrich), supplemented with 10% FBS, 1% Pen-Strep and 2 mM l-glutamine (Sigma-Aldrich). Both cell lines were incubated at 37 °C in a humidified atmosphere containing 5% CO_2_ until ∼90% confluency. All cells were passaged twice within 7 days and then used for amyloid growth and extraction.

To prepare parent wild-type Aβ_40_ (wt-Aβ_40_) or pS8-Aβ_40_ fibrils, purified and lyophilized peptides were first dissolved in hexafluoro-2-propanol (HFIP, Sigma-Aldrich), incubated overnight at ambient temperature, and briefly centrifuged to remove any preaggregates. HFIP was then removed by gentle N_2_ flow followed by 2 h of vacuum drying. The resulting peptide films were dissolved in dimethyl sulfoxide (DMSO) and quantified based on absorbance at 280 nm. The DMSO stock solution was rapidly diluted with 10 mM phosphate buffer (pH 7.4, containing 0.01% *w/v* NaN_3_) to a final Aβ concentration of 10 μM. The volume of DMSO was maintained at <1% (*v/v*) of the total fibrillation solution in all experiments. Samples were incubated quiescently at 37 °C for 14 days to ensure the formation of mature fibrils, as confirmed by negatively stained TEM (Supplementary Figure S1)

Amyloid formation in cells was performed following previously described procedures: Briefly, parent fibrils were probe-sonicated for 2 min (QSonica125, 25% power level, 10 s on/off duty cycle) to generate seeds. The mixture of seeds and freshly dissolved Aβ_40_ monomers in culture medium was added to cells at a 1:10 seed-to-monomer molar ratio and a final monomer concentration of 10 μM. Cells were then incubated for 48 h prior to extraction. The extraction protocol using a sucrose gradient has been described in detail previously (also see Supplementary Figure S1 for TEM images for representative seeds and seeded fibrils).^13^

### SDS-PAGE Quantification

Cell-extracted pellets were first resuspended in 10 mM Tris-HCl buffer (pH 7.4) at a 1:1 (*v/v*) ratio and thoroughly mixed. The mixtures were then diluted with the same buffer and combined 1:1 (*v/v*) with Laemmli sample buffer containing 3.0% (*w/v*) dithiothreitol (DTT, Sigma). A series of 2× diluted solutions was then prepared from this initial stock for gel loading. Samples were heated at 90 °C for 5 min prior to loading onto gels. For each PAGE, a series of diluted solutions of freshly dissolved Aβ_40_ monomers were loaded as an internal standard for quantification. The intensities of Aβ_40_ bands were analyzed using the ImageJ Gels Toolset.

### ThT Fluorescence Kinetics Assay

The ThT fluorescence assay was performed to monitor the elimination of self-nucleated wt-Aβ_40_ fibrillation in the presence of *ex vivo* seeds. Assays were conducted using a multimode microplate reader (BioTek Synergy HTX) with 450 nm/500 nm excitation/emission wavelengths. For each set of experiments, seeds and freshly prepared wt-Aβ_40_ monomers were mixed at 1.0, 2.5, 5.0, and 10.0 mol %. Kinetics traces were recorded for 24–48 h at 37 °C under quiescent incubation, with 10 s of orbital shaking applied before each data collection. Four replicates were performed for each sample condition. All kinetic curves were fitted to the stretched exponential equation below to extract the lag periods^16^

1 $$I_{t}=I_{0}+\left(I_{\infty }-I_{0}\right)\cdot \left[1-\exp\left(-{\left(kt\right)}^{n}\right)\right]$$

where *I*_0_ and *I*_∞_ represent the initial and final fluorescence emission respectively; *k* is the elongation rate constant; and *n* is a power exponent previously applied to describe the kinetics of fractal growth.

### Flow Cytometry

The membrane permeabilities of N2a and SH-SY5Y cells in the presence of self-seeded (10 μM monomeric wt-Aβ_40_ with 1 μM *ex vivo* wt-Aβ_40_ fibrils) and cross-seeded (10 μM monomeric wt-Aβ_40_ with 1 μM *ex vivo* pS8-Aβ_40_ fibrils) Aβ were monitored using flow cytometry. Control cells without peptide addition were also measured. All cells were treated using a commercial Propidium Iodide (PI)/Yo-Pro-1 assay kit (Thermo Fisher) following incubation with Aβ. For each sample, approximately 800,000 cultured cells with various Aβ incubation times were collected by gentle centrifugation (300*g*, 5 min), washed with cold PBS buffer, and stained using PI/Yo-Pro-1 in PBS buffer on ice for 30 min. PI and Yo-Pro-1 fluorescence intensities were recorded using a ZE5 Cell Analyzer (BioRad). Excitation/emission wavelengths were set to 488/509 nm for Yo-Pro-1 and 562/640 nm for PI. Approximately 20,000 events were collected for each sample. Intact and singlet cells were gated for membrane permeability analysis based on their FSC-SSC ratio and FSC area-to-height ratio at 488 nm, respectively. Fluorescence intensities were quantified using FlowJo (BD Biosciences) with a uniform set of Yo-Pro-1/PI intensity thresholds applied across all samples.

### Solid-State NMR Spectroscopy

The current work mainly utilizes ^13^C-PITHIRDs-CT spectroscopy^17^ to quantify the interstrand distances between Aβ peptides at specifically isotope-labeled sites. All ssNMR samples contain Aβ with selectively ^13^C labeled amino acids at three different sites: an Ala residue with ^13^Cβ, a Gly residue with 13Cα and a third type of amino acid (e.g., Val or Phe) with ^13^CO. These ^13^C sites are distinct in their chemical shifts and can be analyzed individually by setting the ^13^C carrier frequencies. All ssNMR measurements were performed on a Bruker Avance III 600 MHz spectrometer equipped with a 2.5 mm TriGamma magic-angle spinning (MAS) probe. Each sample contains ∼3.0 mg of isotope-labeled Aβ aggregates mixed with *ex vivo* extracts from cells.

The following parameters were used for ^13^C-PITHIRDs-CT spectroscopy: a 60 kHz ^1^H π/2 pulse; a 1.5 ms ^1^H–^13^C cross-polarization (CP) with a 65 kHz ^1^H field and a linear-ramped ^13^C field of 45 kHz; and a ^13^C PITHIRDs-CT π pulse train optimized for a 16.7 μs pulse width, matching the 20 kHz MAS spinning frequency. Pulsed-spin locking (PSL) acquisition^18^ was applied with ^13^C carrier frequencies centered at the specific ^13^C sites (e.g., Cβ, Cα or CO). Each PITHIRDs-CT dephasing curve (including seven dephasing times for a single site) was completed with 16–24 h of signal averaging. All spectra were processed with 10 Hz Gaussian line broadening and integrated over 1.0 ppm around the peak for the quantification of ^13^C decay. Fitting of PITHIRDs-CT data will be described in the Results section.

## Results

### The Aβ_40_ Extracts Showed Seed-Type and Cell-Type Specificity in *Ex Vivo* Seeding Efficiency

We previously demonstrated a low-resolution fibrillar architecture for pS8-Aβ_40_ fibrils grown from synthetic peptides in buffer solution.^19^ Compared with wt-Aβ_40_, the pS8 variant’s N-terminal segment folded into the β-sheet hydrophobic cores (i.e., L17-A21 and A30-V36), making it a more effective seed for facilitating the seeded fibrillation of wt-Aβ_40_. We showed that the ThT-based fibrillation rate for pS8-cross-seeded elongation was 8.5-fold higher than that of wt-self-seeded elongation in buffer. Meanwhile, N2a cell toxicity induced by pS8-cross-seeded fibrils was 2-fold higher than that induced by wt-cross-seeded fibrils, as determined by an MTT-based cell viability assay.^20^ Thus, in the current study, we compare the *ex vivo-seeded* elongation of wt-Aβ_40_ fibrils using extracted wt- and pS8-Aβ_40_ amyloids from N2a and SH-SY5Y cells.

Following previously established protocols, we first quantified the concentration of Aβ in the wt-Aβ_40_ and pS8-Aβ_40_ amyloid extracts from the two cell lines. The quantification provided estimated concentrations of Aβ_40_ seeds within the extracted mixtures, which serves as an approximate guidance for the use of monomeric Aβ_40_ concentration in the following ThT kinetics assay. As shown in the representative Figure 1A (also see Supporting Figure S2 for ImageJ quantification), where the SDS gel bands showed differences in intensities. The Aβ_40_ concentrations determined for the batch of cell extracts were 230 μM, 116 μM, 39 μM, and 279 μM for N2a-wt, SH-SY5Y-wt, N2a-pS8 and SH-SY5Y-pS8, respectively. The diverse in seed concentrations was due to the differences in the starting cell populations, and the quantification step was necessary for each individual batches of sample preparation.

**Figure 1:**
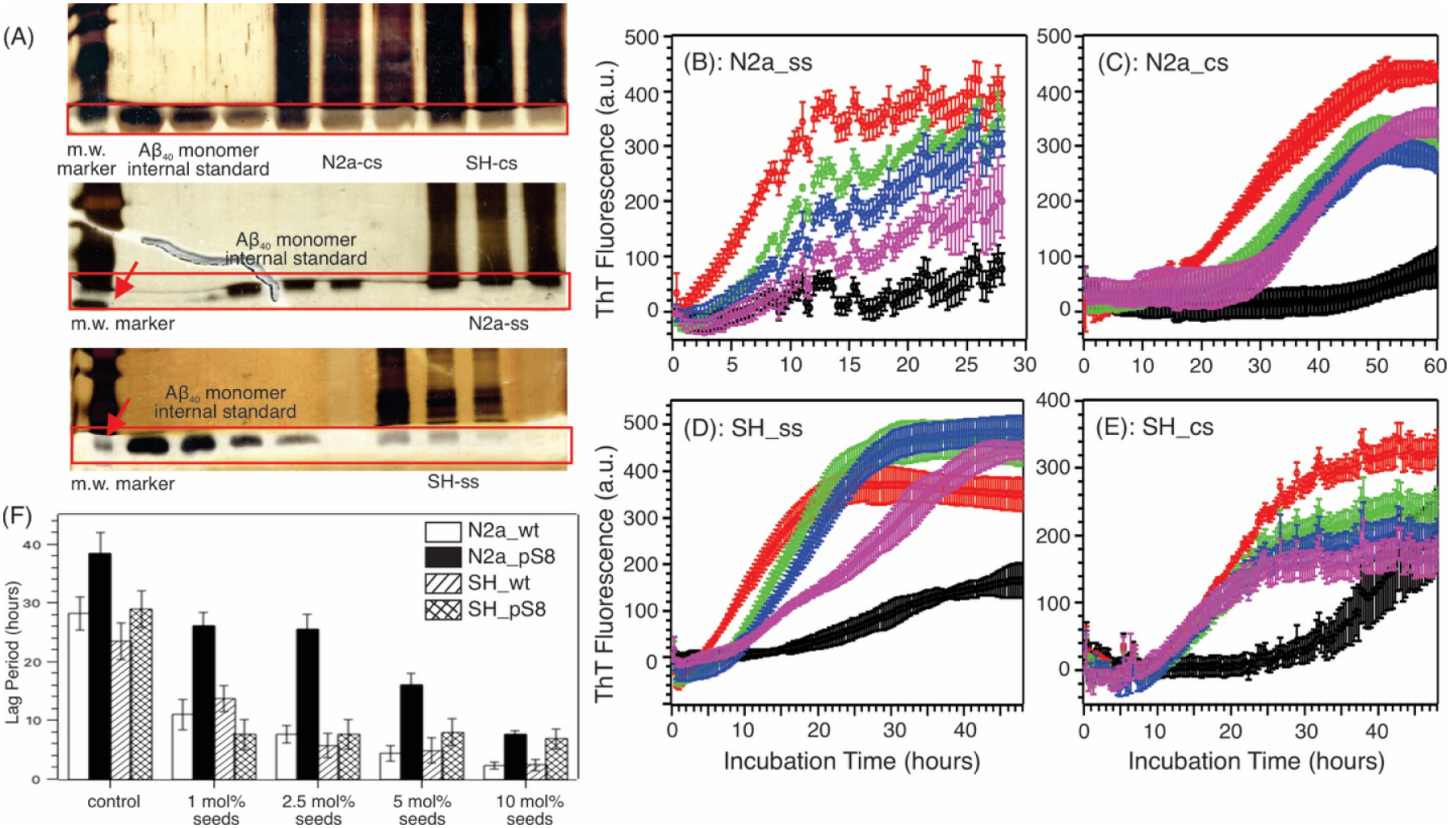
(A) Representative SDS-PAGE bands to quantify the concentrations of Aβ_40_ in *ex vivo* extracts. (B–E) Representative ThT kinetics traces for *ex vivo* seeding. Color-coding: Black, controls with cell-extracts only but no Aβ seeds; Red/Green/Blue/Purple, with cell-extracts contains 10.0/5.0/2.5/1.0 mol % seeds relative to 10 μM fresh wt-Aβ_40_ monomers. Concentrations of seeds were determined by SDS-PAGE. For each set of experiments, the uncertainty bar represents the s.t.d. from four repetitions. (F) Plots of lag periods derived from the fitting of ThT kinetic traces to the stretched exponential equation. Error Bars represent the s.t.d. from fitting of four individual traces.

Given the Aβ concentrations in the extracts, we next determined the optimal seeding times for the ssNMR samples using ThT fluorescence kinetics assays. As shown in Figure 1B–E, the seeded wt-Aβ_40_ fibrillation kinetics were recorded in the presence of four different cell-extracted seeds with various seeds-to-monomer molar ratios (color-coded). For each set of experiments, a control was performed using 10 μM self-nucleated wt-Aβ_40_ monomers in the presence of non-Aβ cell extracts (black traces). Interestingly, the presence of non-Aβ cell extracts also appeared to influence the self-nucleation process, leading to nonuniform lag periods. In the presence of Aβ seeds, the fibrillation was significantly accelerated, as expected, especially with 5 mol % and 10 mol % seeds (i.e., 0.5 μM and 1.0 μM, respectively). Figure 1F shows the best-fit lag periods for all kinetic traces. For the preparation of ssNMR samples, we sought to identify incubation times that allow significant seeded elongation (e.g., reaching or approaching the plateau phase) but precede the onset of self-nucleated elongation. As a result, a 10 mol % seed-to-monomer ratio with 12–36-h incubation times was selected for all samples.

### The Self-Seeded and Cross-Seeded Aggregation of wt-Aβ_40_ Induced Distinct Membrane Permeability Changes in N2a and SH-SY5Y Cells

We monitored the changes in cell membrane permeability using flow cytometry with a double-stained PI/Yo-Pro-1 fluorescence assay. Both dyes fluoresce in dead cells that have disrupted membranes, whereas only Yo-Pro-1 is permeable in early apoptotic cells.^21^ Figure 2A–D display the different cell populations (necrotic/dead cells, Q1 + Q2; apoptotic cells, Q3; live cells, Q4, see Supplementary Table S1 for representative quantifications) for 5 min and 24-h incubations with various seeded fibrillation systems. For quantification, the same gating thresholds were applied to all cell sorting data sets. There were few apoptotic cells in all cases, indicating that the cell deaths were mainly induced by external cytotoxic mechanisms, rather than programmed cell responses.

**Figure 2:**
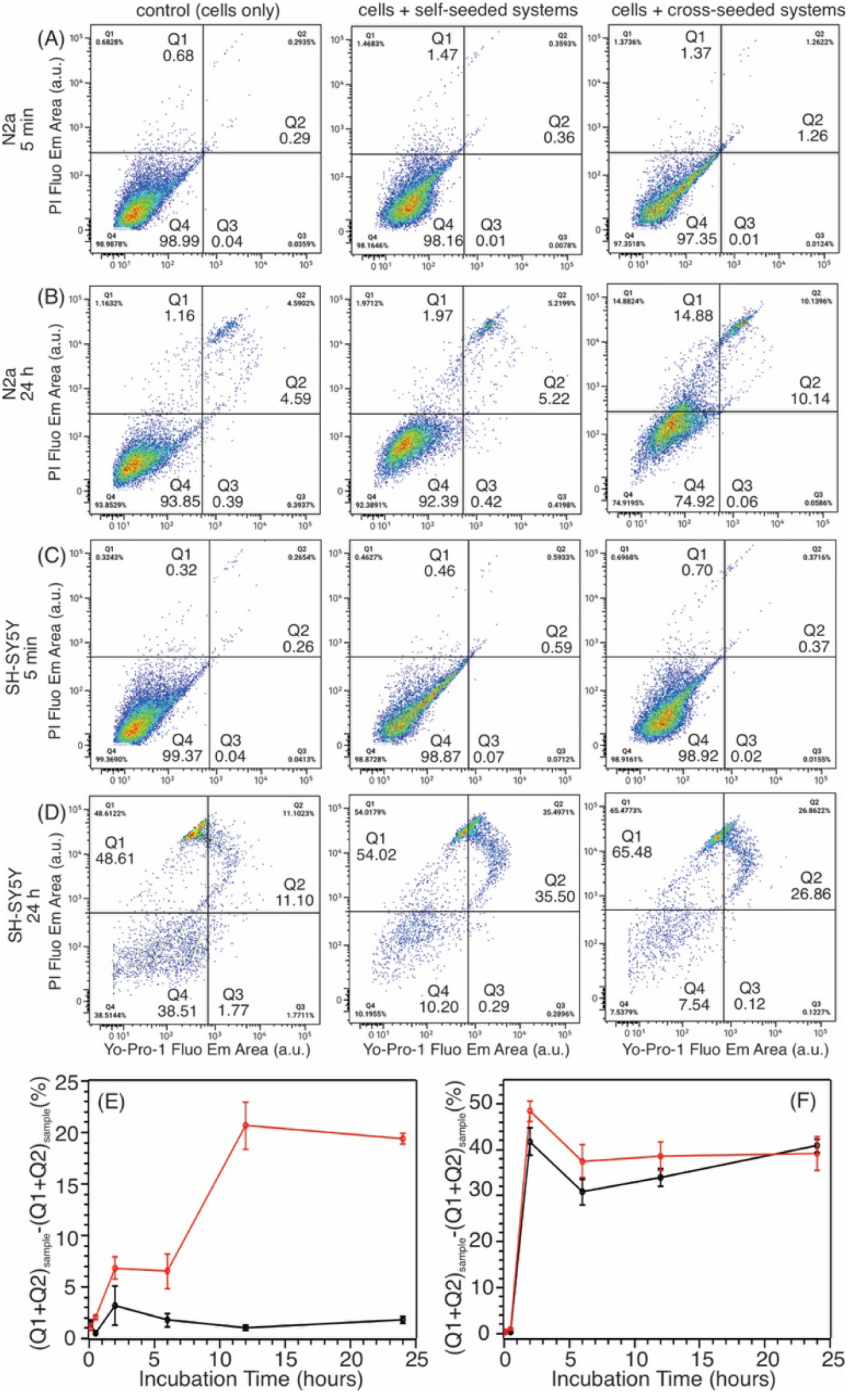
(A–D) Representative plots of cell sorting with 5 min and 24-h incubation times for various seeded fibrillation cellular systems. A uniform division was selected to analyze all sorting outcomes (e.g., the cross drawn in the plots). (E–F) Plots of the increased populations of dead cells as a function of incubation time for N2a (Panel E) and SH-SY5Y (Panel F) cells. Color coding: Black, self-seeding systems relative to corresponding control; Red, cross-seeding systems relative to corresponding controls. Error bars represented the s.t.d. from three repetitions of cell sorting data sets.

The quantitative analyses (Figure 2E–F) indicated that the cross-seeded fibrillation (i.e., wt-Aβ_40_ monomers seeded by pS8-Aβ_40_ *ex vivo* seeds) induced significantly higher N2a cell membrane permeability changes than the self-seeded fibrillation (i.e., wt-Aβ_40_ monomers seeded by wt-Aβ_40_ *ex vivo* seeds). Such differences were modest for the SH-SY5Y cells, which seemed in general less resistant to Aβ aggregation (e.g., more rapid aggregation and higher absolute dead cell populations). We previously showed, using the MTT assay, that the N2a cell viability changed over time in the presence of pS8-cross-seeded fibrillation systems.^20^ The decrease in cell viability only became significant after 24-h incubation, which was slower than the modulations of N2a membrane permeability. Compared with the self-seeded system, the cross-seeded system showed elevated effects on N2a membrane permeability, positively correlated with their relative MTT-based cell toxicity levels. Lastly, the changes in membrane permeability induced by seeded Aβ fibrillation were in general more significant for SH-SY5Y cells.

### Quantitative Analysis of The ^13^C-PITHIRDs-CT ssNMR Data Sets Illustrates Molecular-Level Structural Differences between wt- and pS8-Aβ_40_ Seeds

The molecular-level structural polymorphisms in Aβ fibrils and their pathological relevance have attracted increasing attention. Seeding between various types of amyloidogenic species may contribute to such structural polymorphisms. For instance, it is now considered that coaggregation between Aβ and Tau proteins may produce new pathological polymorphs.^22–24^ Cross-seeding between certain post-translationally modified Aβ variants (e.g., N-pyroglutamate, phosphorylated and nitrated Aβ variants) and wt-Aβ has also been shown to exhibit higher neurotoxicity.^25–31^ In addition, the involvement of cellular environments may further influence the structural propagation and modulation *in vivo*. Here, we focus on the differences in residue-specific interstrand distances, a metric of peptide chain assembly, between the parent seeds and the seeded Aβ fibrils produced with various cell types and *ex vivo* seeds.

We first investigated the interstrand distances in the parent wt-Aβ_40_ and pS8-Aβ_40_ seeds. Nine isotope-labeled sites, namely A2-^13^Cβ, G9-^13^Cα, V12-^13^CO, F19-^13^CO, A21-^13^Cβ, G25-^13^Cα, G29-^13^Cα, A30-^13^Cβ, and V36-^13^CO, were selectively incorporated. Figure 3A-B show the representative ^13^C-PITHIRDs-CT spectra, and the quantification of ^13^C intensity decay curves is plotted in Figure 3C–D (open circles). More rapid decay curves indicate shorter interstrand ^13^C–^13^C distances. It is clear that the wt-Aβ_40_ and pS8-Aβ_40_ seeds were structurally distinct at the residue-specific level. For instance, G9 showed no decay in the wt-Aβ_40_ seeds but rapid decay in the pS8-Aβ_40_ seeds, indicating that the latter fibrils were likely to adopt a β-sheet conformation at this site. In contrast, the interstrand distances at F19 were much closer in wt-Aβ_40_ seeds than in pS8-Aβ_40_ seeds. It is worth noting that each fibril seed may adopt structural polymorphisms, and the experimental ^13^C-PITHIRDs-CT decay represents the ensemble average of such polymorphisms.

**Figure 3:**
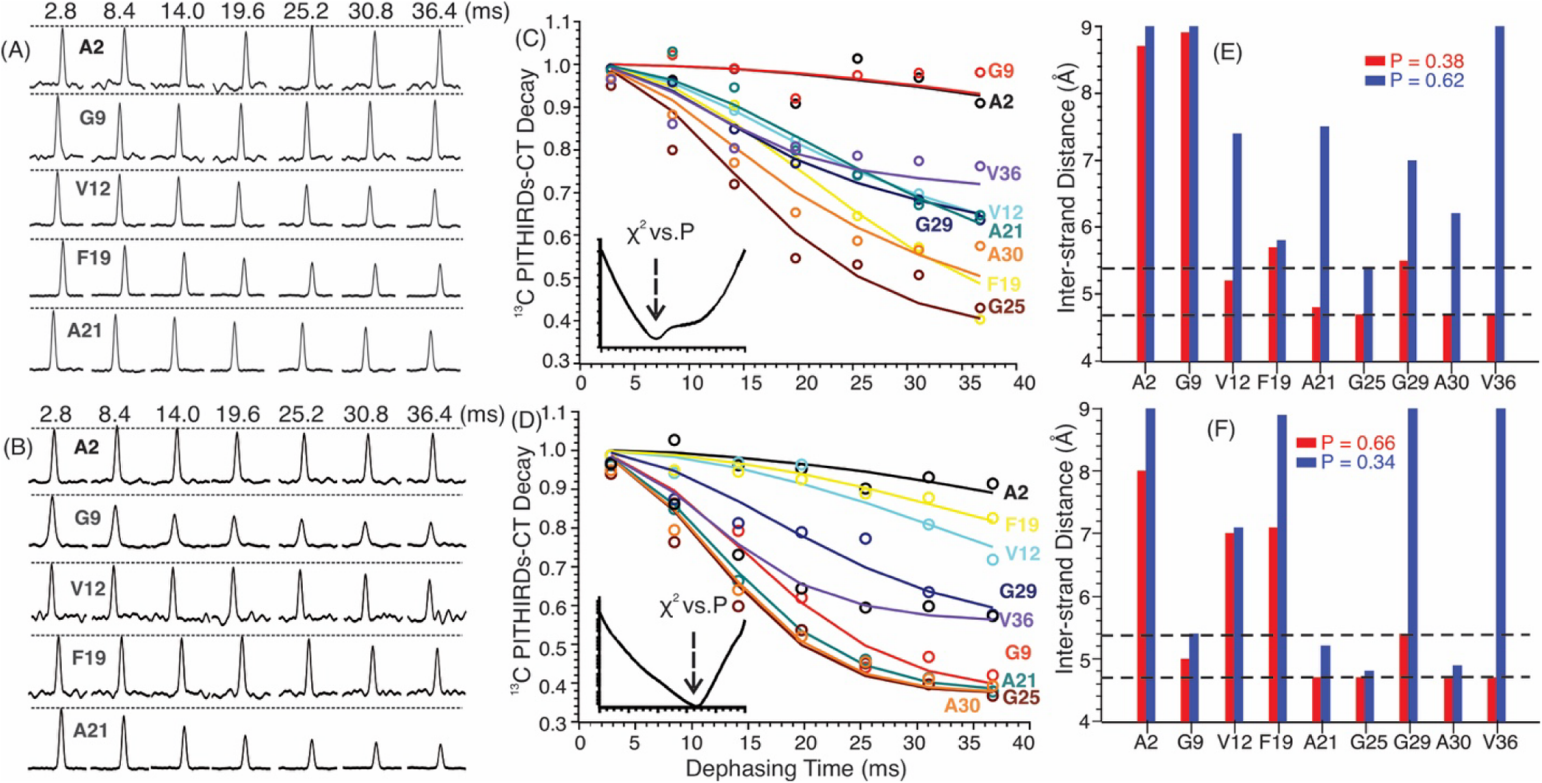
(A–B) Representative ^13^C-PITHIRDs-CT spectra for the parent wt-Aβ_40_ (Panel A) and pS8-Aβ_40_ (Panel B) seeds. Each set of spectra contains seven ^13^C–^13^C dipolar evolution times from 2.8 to 36.4 ms. The decay rate of ^13^C signal is determined by the strength of ^13^C–^13^C dipolar coupling, and therefore the internuclear ^13^C–^13^C distances. All spectra were taken with PSL acquisition, which eliminates the chemical shift information (and therefore not shown). (C–D) Plots of experimental PITHIRDs-CT dephasing (open symbols) and best-fit decay curves (solid lines) for individual residues (color coding highlighted aside of the plots) in wt-Aβ_40_ (Panel C) and pS8-Aβ_40_ (Panel D) seeds. A global two-population fitting was done with minimized χ^2^ as a function of P (“red” population in Panels E–F). (E–F) Plots of best-fit populations and site-specific interstrand distances. The range with two dashed lines indicates parallel-β-sheet, considering the magnitudes of experimental uncertainty.

To quantify the PITHIRDs-CT data, we performed a global fitting for the set of nine decay curves, assuming that there were two structurally distinct populations of fibril polymorphs. A global deviation (χ^2^) between the experimental and simulated PITHIRDs-CT decays was defined as

2 $$\chi^{2}\left(d\right)\;=\underset{i,j}{\sum }\frac{{\left(S_{i,j}^{\exp}\right)}^{2}-[P{\left(S_{i,j}^{\mathrm{sim},1}\left(d\right)\right)}^{2}+\left(1-P\right)\left(S_{i,j}^{\mathrm{sim},2}\left(d\right){)}^{2}\right]}{{\left(\sigma_{i,j}^{\exp}\right)}^{2}}$$

where $S_{i,j}^{\exp}$ is the experimental normalized PITHIRDs-CT data for each residue *i* at each dephasing time *j*. $S_{i,j}^{\mathrm{sim},1}\left(d\right)$ and $S_{i,j}^{\mathrm{sim},2}\left(d\right)$ are the simulated PITHIRDs-CT data sets generated using the SIMPSON program package,^32^ based on a three-^13^C-spin model with a range of internuclear distances *d* from 4.5 to 9.0 Å. $\sigma_{i,j}^{\exp}$ represents the experimental uncertainty derived from the normalized spectral noise, typically in the range of 0.05–0.1 (see the noise levels in Figure 3A–B). The χ^2^ value was minimized as a function of *P*, yielding a global value of *P* and a corresponding set of residue-specific *d* values. The present “two-population” fitting model is a simplified fitting strategy that considers the minimum number of structurally distinct populations in both parent and seeded fibrils. The actual populations of polymorphs may be determined from cryo-TEM 3D classifications and can be used as input values for quantitative ssNMR data fitting. Uncertainties for data fitting can be estimated from the total number of data points (*N* = 63) and the number of independent fitting parameters (*n* = 19). Considering the threshold of one-fold standard deviation of χ^2^ (i.e., $\chi_{\min}^{2}\pm \sqrt{2\left(N-n\right)}$ , the uncertainties are roughly 7–8% of the best-fit *P* values, reflecting the experimental noise levels in the ^13^C-PITHIRDs-CT spectra.

The results of the fitting are illustrated in Figure 3C–D, where the solid lines represent the best-fit simulated decay curves, and the insets display the minimization of χ^2^ as a function of the population *P*. Figure 3E–F shows the best-fit residue-specific distances in the two polymorphs for wt- and pS8-Aβ_40_ seeds. Notably, a 4.8–5.4 Å interstrand ^13^C–^13^C distance (dashed lines) indicates parallel β-sheet assembly at a given site in the current study, considering the spectral noise. The following structural features were noted for the seeds: (1) Both seeds contain a population of more structurally ordered polymorphs and a less-ordered population, but with different weighing factors. The more-ordered structural polymorph populations in wt- and pS8-Aβ_40_ seeds were 0.38 and 0.66, respectively. This is consistent with our previous conclusion that the *in vitro* pS8-Aβ_40_ fibrils possess less dynamic fibrillar cores.^19^ (2) Focusing on the more-ordered polymorphs, the wt- and pS8-Aβ_40_ seeds are structurally distinct. The wt-Aβ_40_ seed typically exhibited non-β-sheet N-terminal sites such as A2 and G9, and typical parallel-β-sheet features for V12, F19, A21, G25, G29, A30 and V36, which agree with other previously proposed wt-Aβ_40_ models.^6,11,33–35^ The pS8-Aβ_40_ seeds, however, exhibited an ordered G9 site and a relatively disordered F19 site.

### Mapping of the Molecular-Level Structural Propagation and Deviation through *Ex Vivo* Seeding

Using the same labeling schemes, we then explored how the residue-specific assembling features propagate through the *ex vivo*-seeded fibrillation (see Supplementary Figure S2 for representative ^13^C-PITHIRDs-CT spectra). Four sets of ssNMR samples were prepared, namely the SHSY5Y-self-seeded (SH_ss), N2a-self-seeded (N2a_ss), SHSY5Y-cross-seeded (SH_cs), and N2a-cross-seeded- (N2a_cs) Aβ_40_ fibrils. All samples were prepared by mixing the monomeric wt-Aβ_40_ peptides with different types of *ex vivo* seeds at optimized seed-to-monomer ratios and seeding times, which were determined by SDS-PAGE and ThT kinetics assays. Figure 4 shows the fitting outcomes of each set of experimental PITHIRDs-CT dephasing curves based on the two-population model. Several conclusions can be drawn: First, it is clear that the molecular-level structural polymorphisms were influenced by both cell and seed types, because the seeded elongation of wt-Aβ_40_, using the same monomeric building block, led to various fibrillar polymorphs. Second, the *ex vivo* seeding tended to produce fibrils that possess more ordered molecular structures. This is supported by two observations. Compared with the parent seeds (Figure 3E–F), all seeded wt-Aβ_40_ fibrils show certain populations that have parallel-β-sheet assembling at most residues (“red” populations in Figure 4). In addition, residues A21, G25, G29, and A30, which are commonly seen in the Aβ_40_ fibrillar core, showed shorter average interstrand distances in seeded fibrils compared with seeds, especially the wt-Aβ_40_ *ex vivo* seeds. Third, certain molecular structural features in the parent seeds are propagated to their daughter fibrils. For instance, we highlighted the distinct features of residues G9 and F19 between the parent wt- and pS8-Aβ_40_ seeds. Figure 4 shows that in self-seeded fibrils (Panels A–B), F19 is more ordered compared with G9; while in cross-seeded fibrils (Panels C–D), G9 is more ordered, recapitulating their features in the parent seeds. Fourth, various *ex vivo* cellular materials may affect the convergence of structural ordering. For seeding in the presence of *ex vivo* SH-SY5Y extracts, a population of fibrils shows well-ordered N-terminal residue A2. For seeding with *ex vivo* N2a extracts, the populations of globally ordered (“red” populations) fibrils increase to more than 50%. Overall, our results demonstrate that, at the molecular level, structural polymorphisms can be induced by the presence of heterogeneous amyloidogenic seeds as well as the involvement of cellular environmental factors.

**Figure 4:**
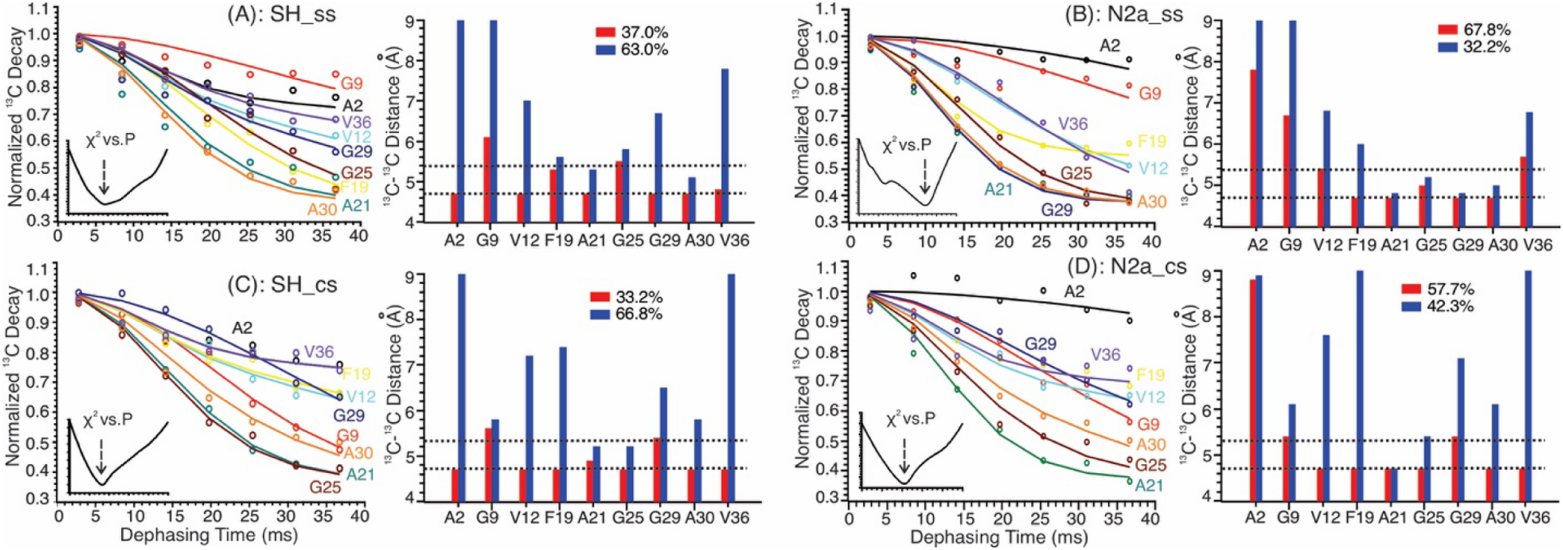
Plots of experimental ^13^C-PITHIRDs-CT dephasing data (open symbols, left panels), best-fit dephasing curves (solid lines, left panels), χ^2^ values as the function of population (insets, left panels), and the best-fit interstrand distances (right panels) for the four *ex vivo* seeded wt-Aβ_40_ fibrils. Individual panels present the four seeding systems with different cell and seed types: (A) SH_ss; (B) N2a_ss; (C) SH_cs; and (D) N2a_cs.

## Discussion

### Implications on the Structural Studies Involving *Ex Vivo* Approaches

It has been recognized that the molecular structures of Aβ fibrils grown *in vitro* differ from those obtained through *ex vivo* approaches,^4,8,35^ and the molecular-level structural polymorphisms of *ex vivo* Aβ fibrils have been shown to be related to disease progression and/or clinical subtypes of Alzheimer’s patients.^5,9^ Establishing correlations between fibrillar structural polymorphisms and AD progression/clinical subtypes, if achieved, would play a significant role in both diagnosis and treatment. As a predominant approach currently used to address this question, single-particle cryo-TEM classifies fibrillar polymorphs based on their morphological features, such as helical twisting patterns, given the availability of a large number of high-quality micrographs. *Ex vivo* cryo-TEM uses samples that are directly extracted from biological specimens, therefore introducing minimal perturbation to the amyloid structures during sample handling. However, challenges exist when using cryo-TEM alone to explore potential correlations between fibrillar structural polymorphisms and disease-related metrics. For example, certain fibrillar polymorphs without significant morphological features (e.g., untwisted filaments) may be underestimated due to current limitations in cryo-TEM data processing.^36^ In addition, structural polymorphs with less-ordered core segments may also be underestimated because of their intrinsically lower resolution in cryo-TEM-based 3D structural reconstruction.

The current study indicates that quantitative ssNMR may provide a complementary approach to support or verify the cryo-TEM screening of fibrillar structural polymorphisms. Theoretically, the distributions of structural polymorphs should be reflected in the classifications of cryo-TEM micrographs, as well as in the ensemble-average of quantitative ssNMR measurements, given that the morphological features and molecular structures of fibrils are closely related.^3,33,37–39^ Therefore, the population distributions obtained by cryo-TEM and quantitative ssNMR measurements should also be related. A population distribution obtained by cryo-TEM for the parent or seeded fibril can be introduced to the fitting of quantitative ssNMR to the same fibril (i.e., to set an appropriate range for the global population parameter *P*). If the fitting only focuses on specific structural features of interest, for instance, the average interstrand distance at a specific site as given in the present study, the obtained structural parameters from ssNMR can be used to validate the same parameter derived from cryo-TEM-based structures. Furthermore, if the solution of cryo-TEM-derived structures at the interested site is insufficient (e.g., the site is located in a disordered segment), the ssNMR-based structural information can be complementary.

An implication from the current study is that the combination of cryo-TEM and guided quantitative ssNMR spectroscopy may be used to track the seeding-induced fibrillar polymorphisms. However, it is important to choose specific sites for such tracking. This is because molecular level structural perturbation can be introduced during the *ex vivo* seeding, even when the seeding parameters, such as concentrations and incubation times, have been well controlled (i.e., self-nucleated fibril growth has been minimized). Comparisons between the distribution of interstrand distances in the parent (Figure 3) and seeded (Figure 4) fibrils clear demonstrated that not all residues have their molecular-level structure propagated during this seeding procedure. Residues G9, V12, F19, G25 and V36 are among the residues that may be used for such purposes, because their site-specific assembly features are largely conserved through seeding, and the outcomes appear insensitive to cell type. Other tested residues such as A21, G29 and A30, showed a general tendency to become more assembled and ordered through seeding. Several *ex vivo* wt-Aβ_40_ fibril structures were reported previously,^8,40,41^ which showed discrete β-strands connected by turn-like structural motifs (Figure 5). It is worth noting that the conserved residues in current works are mostly located within the β-strand motifs in these structural models. On the contrary, the nonconserved residues are likely to be in the turn motifs. The differences in their local secondary structures might influence their distinct structural propagation features through seeding.

**Figure 5:**
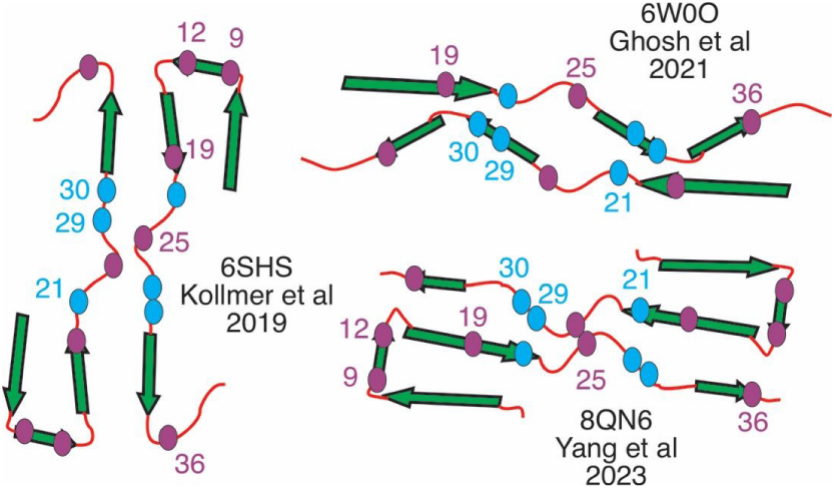
Mapping of structurally conserved residues (G9, V12, F19, G25 and V36, in purple) and modulated residues (A21, G29 and A30, in cyan) onto representative *ex vivo* Aβ_40_ fibril structures. The segments of β-strands are shown in green arrows.

### Implications on the Biological Relevance of Seeding-Induced Structural Polymorphisms

Although the molecular-level structural differences between *in vitro* and *ex vivo* Aβ fibrils have been demonstrated by accumulating evidence, the origin and biological significance of such differences remain unclear. Our current results support the hypothesis that seeding with other Aβ variants or amyloidogenic proteins may contribute to such molecular structural modulations. The pS8-Aβ_40_ is a pathologically relevant post-translationally modified Aβ variant, which was found in early stage amyloid plaques in previous immunochemical studies.^19,27,29,42,43^ This variant has been shown to accelerate the accumulation of wt-Aβ through seeding and increase neurotoxicity.^20,29^ We also showed in the present study that cross-seeding with *ex vivo* pS8-Aβ_40_ led to more significant disruption of neuronal cell membranes compared with self-seeding with wt-Aβ_40_. Accumulation of amyloidogenic proteins in human brain may involve contributions from a heterogeneous pool of compositions, where the effective seeds that form earlier may modulate the structures of aggregates later through seeded fibrillation. We showed here that such structural modulation occurred at the residue-specific level and may be classified into two categories:

First, for the residues that are located within the fibrillar core (e.g., A21, G25 and A30), the seeding seems to result in a more ordered assembly in general. The molecular-level structural features in parent seeds do not propagate into daughter fibrils. In the present work, the parent *ex vivo* pS8-Aβ_40_ seeds showed shorter interstrand ^13^C–^13^C distances at the A21, G25 and A30 sites compared with the wt-Aβ_40_ seeds. However, such differences were not observed in their corresponding daughter fibrils. Biologically, this feature suggests that the fibrillar core in senile plaques may become more rigid at later stages of accumulation compared with earlier stages. Therefore, it may be feasible to utilize this physicochemical metric to assess the progression of amyloidosis.

Second, among the conserved residues, G9 and F19 showed characteristic structural features that were propagated from their parent seeds. Our results suggest that such molecular-level structural propagation may occur in various cellular environments (e.g., in the presence of *ex vivo* extracts from both N2a and SH-SY5Y cells). It is possible that the site specificity of such propagation is determined by the types of variants. For instance, the presence of phosphorylated S8 may facilitate the formation of a stable local structural domain involving the neighboring G9, which is sustained through cross seeding with wild-type Aβ monomers. For a different type of Aβ variant, or other amyloidogenic sequences with cross seeding efficiency, the propagated features/sites may vary. In this case, such propagated structural features may serve as fingerprints to track the origin of structural polymorphisms in senile plaques. Computational tools that can predict long-range interactions in amyloidogenic aggregates based on primary sequences might help to identify such potential fingerprint structural features.

## Conclusion

In conclusion, the current study showed that the *ex vivo* seeding conditions, especially the seed-to-monomer ratio and incubation time, can be optimized for the cross-seeding of Aβ fibrillation to eliminate the primary nucleation. Quantitative ssNMR spectroscopy demonstrated molecular level structural propagation and modulations along the seeded fibrillation process. For the cross-seeding induced by the S8-phosphorylated Aβ_40_ in cells, certain structural restraints such as the interstrand distances at G9 and F19, may be used as characteristic features to monitor the molecular structural propagation from the parent seeds to the seeded fibrils. Future work will address the minimization of secondary nucleation in the *ex vivo* seeding.

## Supplementary Material



## Notes

Additional negatively stained TEM images for parent fibrils, seeds and seeded fibrils, representative SDS-PAGE gel quantifications, and supplementary ^13^C-PITHIRDs-CT spectra (PDF)
