# Supplementary material for: Controlled Seeding of β‑Amyloid Fibrillation Reveals Propagation of Structural Polymorphisms in Cellular Environments
Source: Biochemistry. 2026 Apr 6;65(8):1261–9. doi: 10.1021/acs.biochem.6c00013 (PMC13104022; doi:10.1021/acs.biochem.6c00013)
Supplement: Supplementary file 1 [file bi6c00013_si_001.pdf]

Supplementary Information

Controlled Seeding of β-Amyloid Fibrillation Reveals Propagation of Structural Polymorphisms in Cellular Environments

Tingyao Wang[1], Yan Sun[2], Yi Ran Lin[1], Lan Yao[2], and Wei Qiang[1*]

[1]Department of Chemistry, Binghamton University, the State University of New York, Vestal, New York 13850
[2]Small Scale System Integration and Packaging (S3IP), Binghamton University, Vestal, New York 13850

Corresponding Author: Dr. Wei Qiang (Email: wqiang@binghamton.edu)

Supplementary Figures

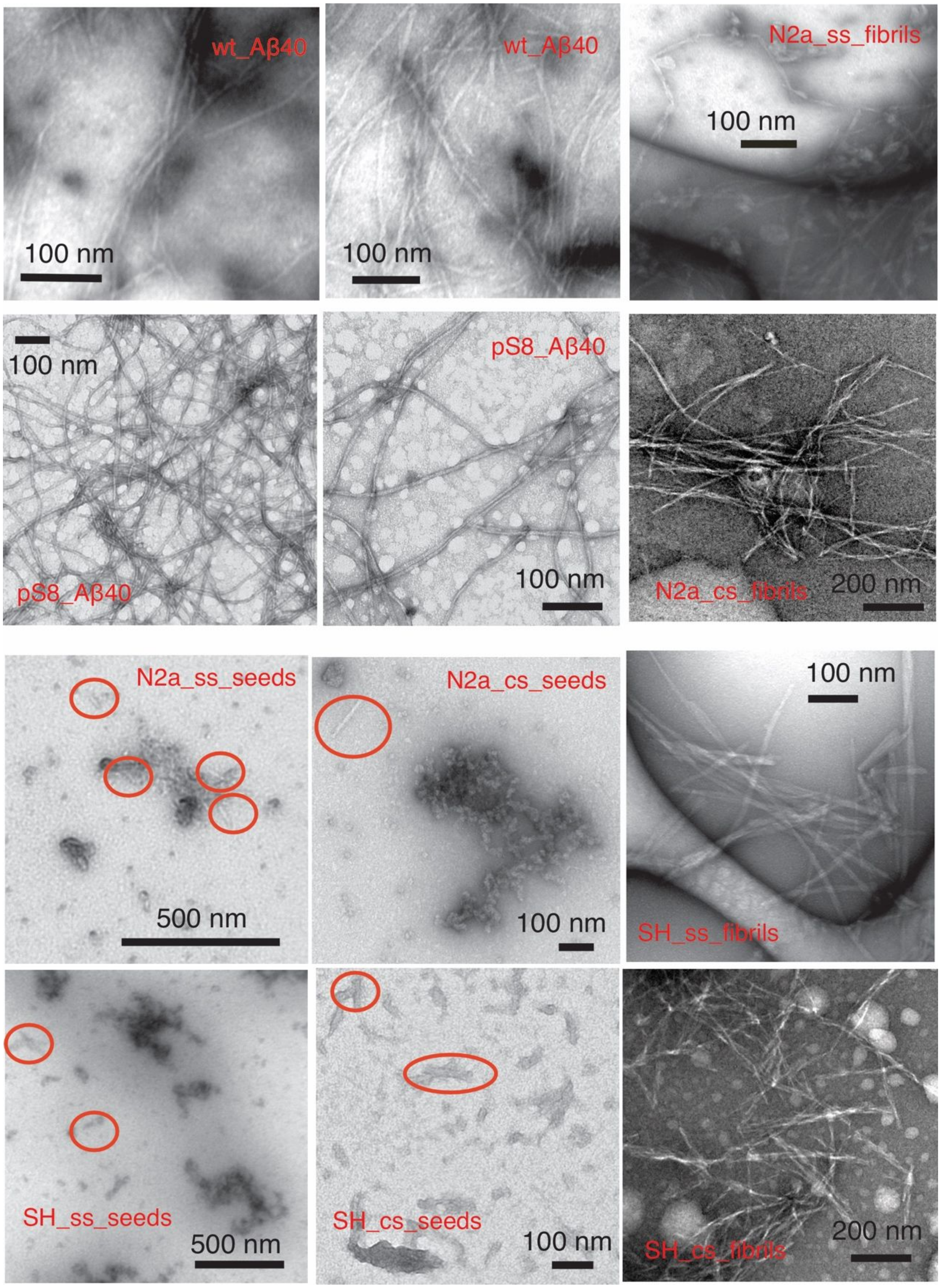

Figure S1 Representative negatively stained TEM images for the parent wt- and pS8-Aβ_{40} fibrils, four types of seeds (N2a_ss, N2a_cs, SH_ss and SH_cs) and seeded fibrils.

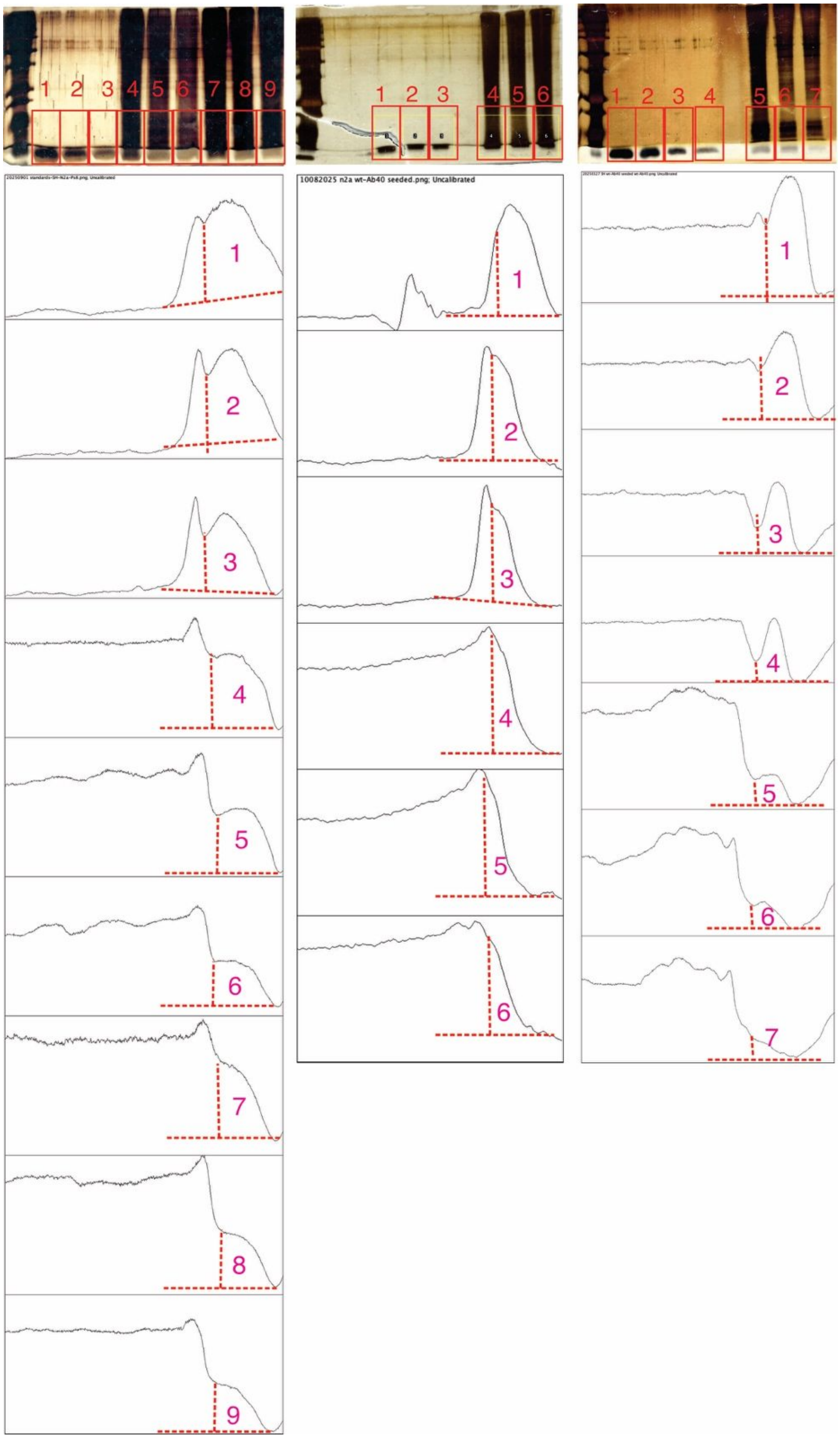

Figure S2 Representative SDS Gel images and ImageJ analyses of SDS-PAGE gel band intensities for (left) N2a-cs and SS-cs; (middle) N2a-ss; and (right) SH-ss.

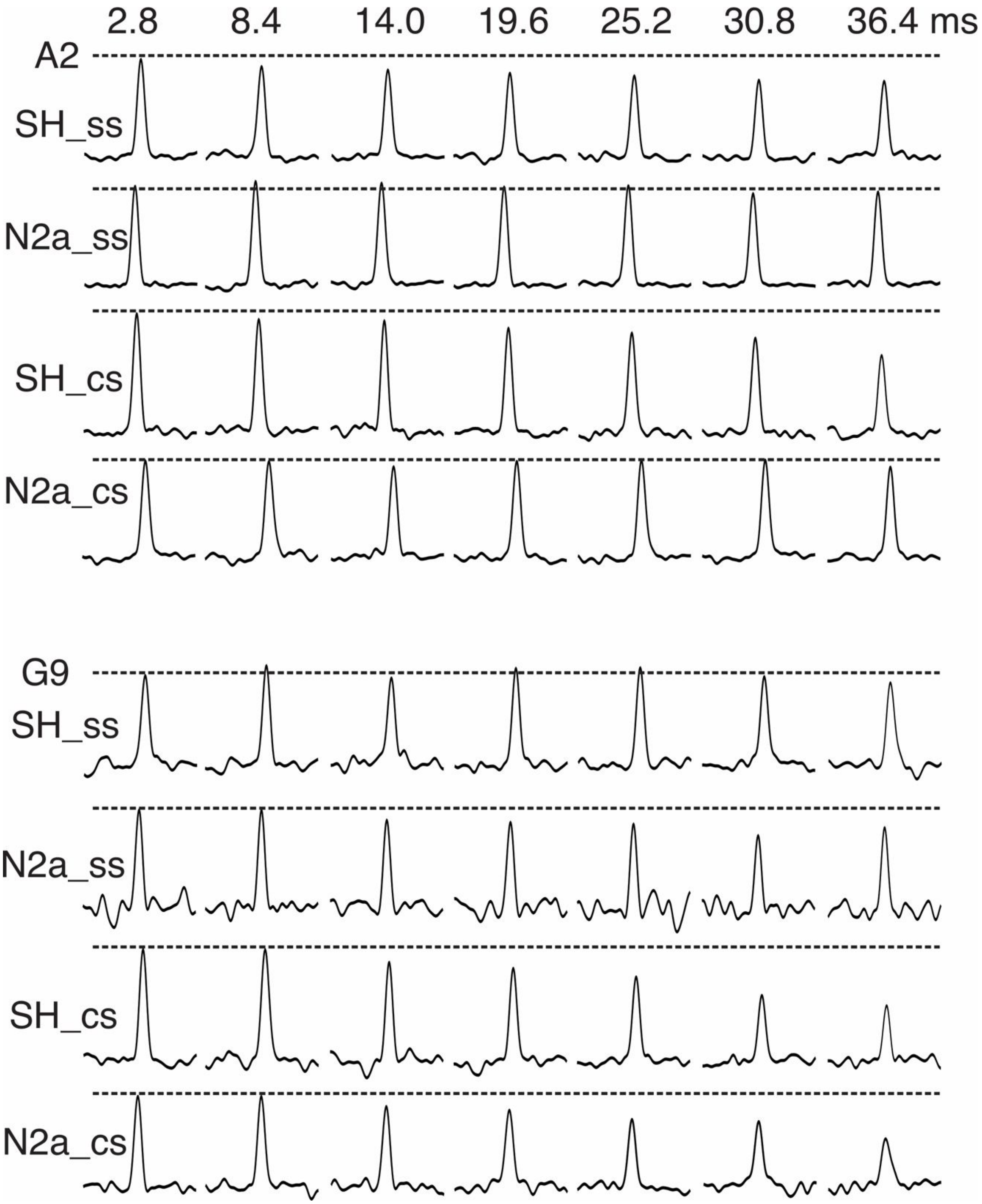

Figure S3 Representative ^{13}C-PITHIRDs-CT spectra for A2 and G9 in four different seeded wt-$A\beta_{40}$ fibrils.

Supplementary Table S1

Quantification of sorted cell populations treated with seeded fibrillation systems for N2a cells.

Samples	Time (hrs)	Q1	Q2	Q3	Q4
N2a_blank_1	0.083333333	0.6828	0.2935	0.0359	98.9878
N2a_blank_2	0.083333333	0.6263	0.3439	0.0246	99.0053
N2a_blank_3	0.083333333	0.4833	0.4413	0.042	99.0334
N2a_self_1	0.083333333	1.3736	1.2622	0.0124	97.3518
N2a_self_2	0.083333333	1.4487	1.1192	0.0124	97.4196
N2a_self_3	0.083333333	0.9858	1.0918	0.131	97.7914
N2a_cross_1	0.083333333	1.4683	0.3593	0.0078	98.1646
N2a_cross_2	0.083333333	1.6305	0.2923	0	98.0772
N2a_cross_3	0.083333333	1.7854	0.4999	0	97.7147
N2a_blank_1	0.5	1.1488	0.5144	0.0343	98.3025
N2a_blank_2	0.5	1.3549	0.5304	0.0807	98.0339
N2a_blank_3	0.5	1.4467	0.5425	0.0329	97.978
N2a_self_1	0.5	2.017	0.4126	0.0917	97.4788
N2a_self_2	0.5	2.0459	0.3701	0.0822	97.5018
N2a_self_3	0.5	1.8394	0.5229	0.1969	97.4408
N2a_cross_1	0.5	3.2476	0.479	0.2184	96.0549
N2a_cross_2	0.5	3.548	0.4561	0.2242	95.7718
N2a_cross_3	0.5	3.3662	0.5884	0.5473	95.4981
N2a_blank_1	2	8.7558	2.0976	0.0556	89.0911
N2a_blank_2	2	10.01	2.2119	0.0625	87.7156
N2a_blank_3	2	10.2134	2.3538	0.0551	87.3778
N2a_self_1	2	11.707	1.9014	0	86.3915
N2a_self_2	2	14.4391	2.4557	0	83.1052
N2a_self_3	2	12.3368	2.4167	0.0195	85.2271
N2a_cross_1	2	15.6232	2.4443	0.0858	81.8468
N2a_cross_2	2	16.2791	2.6181	0.0658	81.037
N2a_cross_3	2	16.7332	2.4614	0.0376	80.7678
N2a_blank_1	6	2.6765	3.842	0.7802	92.7012
N2a_blank_2	6	2.5933	4.7355	0.943	91.7282
N2a_blank_3	6	3.5637	3.9497	0.8315	91.6551
N2a_self_1	6	3.3544	5.8755	1.192	89.5781
N2a_self_2	6	3.4618	5.107	0.8175	90.6136

N2a_self_3	6	3.7118	5.1715	1.0531	90.0636
N2a_cross_1	6	6.8698	4.9694	0.5242	87.6365
N2a_cross_2	6	7.7058	6.3122	0.445	85.5369
N2a_cross_3	6	7.6504	7.3904	0.6066	84.3526
N2a_blank_1	12	1.1915	3.0625	0.3258	95.4203
N2a_blank_2	12	0.9835	3.5806	0.3533	95.0826
N2a_blank_3	12	1.108	3.3626	0.3661	95.1633
N2a_self_1	12	0.8316	4.4417	0.7099	94.0168
N2a_self_2	12	0.9835	4.5806	0.3533	94.0826
N2a_self_3	12	0.8402	4.7996	0.6277	93.7325
N2a_cross_1	12	5.8327	17.0918	0.1524	76.9231
N2a_cross_2	12	5.667	21.811	0.1815	72.3404
N2a_cross_3	12	6.2296	18.6127	0.174	74.9837
N2a_blank_1	24	1.1632	4.5902	0.3937	93.8529
N2a_blank_2	24	1.0844	4.6192	0.3586	93.9378
N2a_blank_3	24	0.8933	4.3159	0.3803	94.4105
N2a_self_1	24	1.9712	5.2199	0.4198	92.3891
N2a_self_2	24	2.0017	5.3806	0.3581	92.2597
N2a_self_3	24	2.1941	5.296	0.4729	92.0371
N2a_cross_1	24	14.8824	10.1396	0.0586	74.9195
N2a_cross_2	24	14.3668	10.1617	0.0674	75.4042
N2a_cross_3	24	15.2951	10.0111	0.0506	74.6432
